# Influence of occupation on the subjective health of forced migrant doctors-a qualitative study

**DOI:** 10.1093/eurpub/ckac130.114

**Published:** 2022-10-25

**Authors:** V Locher, A Krämer

**Affiliations:** School of Public Health, Bielefeld University, Bielefeld, Germany

## Abstract

**Background:**

With the refugee movement in 2015, also forced migrated female and male medical professionals have arrived in Germany. The effect of occupation on the subjective health status of these physicians working in the German health care system was investigated on the basis of Antonovsky's sense of coherence (SOC) and the occupational science models of Siegrist and Karasek&Theorell.

**Methods:**

Using a semi-structured interview guide, nine forced migrated physicians were interviewed before and nine forced migrated physicians were interviewed during their professional medical activity. Both interview groups had an Arabic cultural background. The transcribed interviews were analyzed according to Kuckartz's content structuring qualitative content analysis using the MAXQDA 2020 software tool.

**Results:**

The SOC of migrated physicians is favorably influenced by meaningful occupational activity and the newly gained manageability of life. Positive influences are seen in professional appreciation and collegial support at all hierarchical levels. Negative effects are perceived in experiences of discrimination, insecurity and experienced injustice in the recognition of foreign qualifications. Physical stress results from occupational overload, unfamiliar work and time pressure.

**Conclusions:**

The salutogenic effect of the work, the recognition in the profession and the collegial support are essential contributions to the promotion of especially mental health among forced migrated doctors. This speaks in favor of rapid and stringent integration into professional life. However, organizational barriers inherent in the German health care system should not be disregarded, which is why both legal and structural improvements should be made to the existing integration procedure before and during professional activity.

**Key messages:**

- Expediting the integration of migrant doctors back into their professions is of salutogenic importance.
- Therefore, coordinated and corrective measures should be taken to this end by those responsible for this process.

